# Myopia as a Global Public Health Challenge a Narrative Review

**DOI:** 10.3390/life16071047

**Published:** 2026-06-24

**Authors:** Francesca-Cristiana Dohotariu, Nicoleta Anton, Valeria Coviltir, Vasile Potop, Irina-Andreea Pavel, Ștefan Tudor Bogdănici, Camelia Margareta Bogdănici

**Affiliations:** 1 Doctoral School, Grigore T. Popa University of Medicine and Pharmacy, University Street No. 16, 700115 Iasi, Romania; francesca.dohotariu@gmail.com; 2 Department of Ophthalmology, Faculty of Medicine, Grigore T. Popa University of Medicine and Pharmacy, University Street No. 16, 700115 Iasi, Romania; andreea.niagu@umfiasi.ro (I.-A.P.); tudor.bogdanici@gmail.com (Ș.T.B.); camelia.bogdanici@umfiasi.ro (C.M.B.); 3 Ophthalmology Department, “Carol Davila” University of Medicine and Pharmacy, 050474 Bucharest, Romania; valeriacoviltir@yahoo.com (V.C.); potopvasile@yahoo.com (V.P.)

**Keywords:** pathologic myopia progression, myopia risk factors, pathologic myopia genes

## Abstract

(1) Background: The global prevalence of myopia has increased substantially in recent decades. Myopia development is influenced by both environmental factors and a complex genetic architecture involving more than 400 susceptibility loci. The interaction between genetic predisposition and environmental exposures plays a critical role in myopia onset and progression. Unequal access to preventive strategies and eye care services continue to limit effective global myopia control. (2) Methods: This structured narrative review synthesizes evidence identified through systematic database searches, manual reference screening, prospective cohort studies, randomized controlled trials, mechanistic investigations, and genetic analyses identified through the literature search. (3) Results: Environmental factors such as limited outdoor activity, intensive near-work, and academic pressure contribute to myopia progression. Key biometric indicators, such as AL, AL/CR ratio, and choroidal thinning, are strong predictors. Molecular and cellular mechanisms also contribute significantly to myopia progression. Genetics also plays a significant role, with both syndromic and polygenic pathways involved. (4) Conclusions: As precision medicine evolves, individualized therapeutic strategies are becoming important in myopia management. In the treatment of myopia, biomarkers, genetic profiling, and artificial intelligence may support personalized risk assessment and treatment decisions.

## 1. Introduction

Myopia is commonly defined as a spherical equivalent refractive error of ≤−0.50 D and is primarily associated with excessive axial elongation of the eye. Although myopia was previously considered predominantly an East Asian public health issue, recent European data now clearly indicate a steady rise in its occurrence among Westerners over the last five decades [1]. Experts forecast that by 2050, nearly half of the world’s population (49.8%) will be myopic, and a substantial percentage of them (9.8%) will develop severe forms [2].

When encountering cases of advanced myopia, defined by a prescription of −6.00 diopters or an axial length exceeding 26.5 mm, significant consideration should be given to the high incidence of complications, including retinal detachment, myopic macular neovascularization, glaucoma, cataracts, and posterior staphyloma [3].

Myopia is classified based on spherical equivalent (SE) refractive error and axial length (AL). Clinically, low myopia is typically defined as an SE ranging from −0.50 D to −6.00 D, while high myopia is marked by an SE of at least 6.00 D and is often linked to significant axial elongation. While SE continues to be the standard refractive measure, AL offers a structural evaluation of eye development and may more accurately indicate the danger of myopia-related alterations. The ratio of axial length to corneal radius (AL/CR) has been suggested as an effective biometric measure for differentiating various levels of myopia [4].

A distinction must be made between the onset and the progression of myopia. Myopia onset signifies the shift from emmetropia or premyopia to a myopic refractive condition, usually defined by a SE of ≤−0.50 D. Premyopia, characterized by refractive values close to emmetropia, represents a critical stage during which biometric and environmental risk factors contribute to the development of myopia. Early identification of children at risk during this stage may facilitate preventive measures before the development of permanent refractive error [4].

In contrast, the progression refers to the worsening of an existing myopic state due to rising negative SE values and ongoing axial elongation as time passes. Research indicates that growth in axial length is a crucial structural indicator of progression and might occur before or alongside refractive changes [4].

Early detection of myopia is essential for reducing the risk of progression to high myopia and its associated complications, highlighting the importance of early intervention in preventing progression [4].

When evaluating the risk of a child developing myopia, several factors must be considered, including mild hyperopia, specific ratios of axial length to corneal curvature, and lifestyle habits that may classify the patient as pre-myopic, a newly recognized refractive stage. Early identification of premyopia may improve clinical outcomes by enabling timely preventive interventions before significant axial elongation occurs [4].

## 2. Materials and Methods

This structured narrative review synthesized findings from systematic database searches, manual reference screening, prospective cohorts, randomized trials, mechanistic studies, and genetic analyses.

### 2.1. Database Searches

Studies published between January 2018 and March 2026 were identified through systematic searches of PubMed, Scopus, and Web of Science. The literature search strategy incorporated the following keywords and keyword combinations: “pathologic myopia progression”, “myopia risk factors”, and “pathologic myopia genes”, “Artificial Intelligence and Myopia Management”, “myopia progression genes or myopia genes”.

### 2.2. Eligibility Criteria

Studies published after 2018 in English or other languages were considered eligible for inclusion. Inclusion criteria: Articles published between January 2018 and March 2026 were assessed for relevance to the epidemiology, pathophysiology, biomarkers, genetics, monitoring strategies, and therapeutic interventions associated with myopia. Conference abstracts, posters, computational simulations, and Letters to the Editor were excluded. Due to methodological heterogeneity among included studies, a narrative synthesis approach was considered more appropriate than a formal quantitative meta-analysis.

### 2.3. Study Selection

Studies meeting the eligibility criteria were initially screened based on their titles and abstracts. Subsequently, full-text articles underwent detailed evaluation. Any discrepancies regarding study eligibility or interpretation were resolved through discussion and consensus between two reviewers (N.A. and F.-C.D.).

## 3. Results

**Figure 1:**
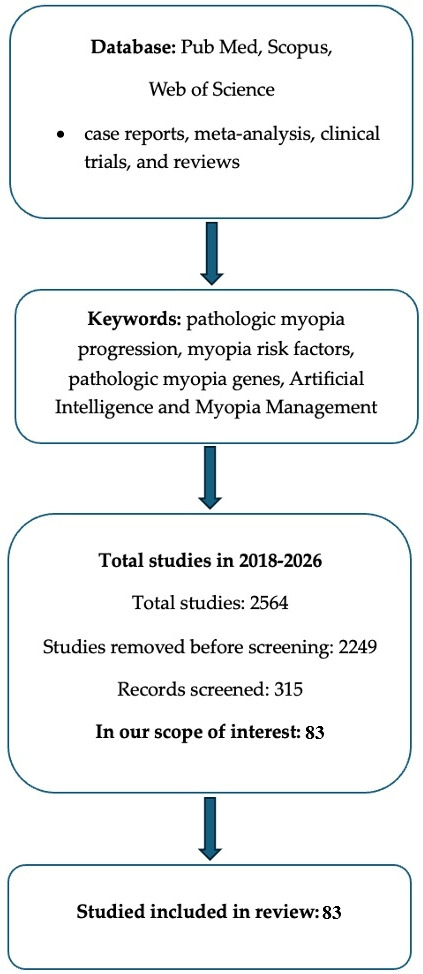
Flow diagram illustrating the study identification, screening, eligibility assessment, and inclusion process. A total of 83 studies were included in the final narrative synthesis.

The literature search identified a total of 2564 records across the selected databases. After the removal of 114 duplicate records and 907 records that did not meet the eligibility criteria, the remaining studies underwent further screening and evaluation. Following the application of the inclusion and exclusion criteria, 83 studies were included in the final narrative synthesis. The study selection process is summarized in Figure 1.

### 3.1. Epidemiology

#### 3.1.1. Regional and Global Prevalence Trends

Current epidemiological evidence indicates that the global prevalence of myopia continues to increase, and substantial regional variations remain. Current modeling studies project that nearly half of the global population will be myopic by 2050, with approximately 10% developing high myopia, defined as a refractive error of at least −5.0 D or an axial length of ≥26.5 mm [1]. Parental myopia significantly increases the risk of developing myopia (OR: 1.21–1.36), consistent with established patterns of genetic heritability [5,6].

Several studies have reported that individuals with at least one myopic parent exhibit age-related alterations in corneal biomechanical properties during follow-up. These changes may reflect altered ocular biomechanics associated with axial elongation and myopia progression. Although this decline was theoretically anticipated with aging, the authors suggest that variation in these eyes might be linked to altered biomechanical stress distribution in the posterior segment, which could influence ocular elongation [7,8].

Although East and Southeast Asia remain the regions most affected by the myopia epidemic, new evidence from the European population-based studies shows clear secular increases, challenging earlier assumptions that Western populations would remain relatively unaffected by the increasing incidence of childhood myopia [9].

A notable retrospective analysis of the Swedish male conscripts aged 17–19 years revealed an increase in overall myopia prevalence from 22% in 1975 to 29% in 1995, with high myopia almost doubling during the period mentioned above [10].

A meta-analysis of Singaporean studies found that three genetic loci were strongly associated with myopia in highly educated individuals but showed only weak associations in those with lower education. The rise in myopia was strongly associated with educational achievement and cognitive performance, indicating that Western myopia trends are driven by mechanisms similar to those observed in Asia [6].

Similar secular increases have been documented in Austria, Israel, and parts of Northern Europe using standardized refractive definitions, highlighting that urbanization and increasing educational demands are global factors rather than confined to specific regions [6].

#### 3.1.2. Age of Onset and Sex Differences

Recent studies involving European and Asian populations suggest that the age at myopia onset may have a greater influence on long-term visual outcomes than the subsequent rate of progression. An earlier age of myopia onset has been more frequently associated with a higher rate of complications and poor visual outcome over time. Children who develop myopia before the age of 8 years generally exhibit greater axial lengths during adolescence [11].

Current research has also revised sex-specific findings. According to older studies, myopia was found to be more common in males. However, recent data gathered from multi-country European cohorts show a higher prevalence and faster progression in females. This difference may be associated with variations in educational exposure and near-work activities [12].

#### 3.1.3. Educational Exposure and Cognitive Correlates

Recent multivariable analyses have further strengthened the association between educational exposure and myopia development. Data from Swedish conscripts revealed that myopia is most strongly associated with attending theoretical upper secondary education (OR ≈ 1.7), followed by cognitive scores assessed through verbal, visual, and spatial stimuli [10]. These findings align with the recent multinational studies suggesting that education influences the expression of genetic risk, thereby increasing polygenic susceptibility in environments with high academic demands. Cognitive ability itself is unlikely to represent the primary causal factor; greater emphasis should be placed on prolonged near-work activity and reduced outdoor exposure [13].

#### 3.1.4. Public Health Implications

The rising prevalence of myopia in Europe and Western regions contradicts earlier forecasts of stabilization. Many analyses emphasize the critical importance of implementing preventive measures at the population level, particularly for young children [2,9].

National pediatric screening strategies are increasingly needed to address the growing burden of myopia. Studies recommend integrating myopia prevention into national eye health and educational policies. Such strategies may facilitate earlier detection of risk factors and improve preventive interventions. Without coordinated public health interventions, healthcare systems may face a substantial increase in the burden of myopia-related complications. More emphasis should be placed on proactive prevention, early screening, and equitable access to control measures to lessen the long-term public health impact of the myopia epidemic [9].

### 3.2. Determinants of Myopia Development and Progression

#### 3.2.1. Environmental and Behavioral Determinants

##### Outdoor Exposure: Dose-Response Evidence

A 2025 systematic review and meta-analysis of randomized controlled trials demonstrated a dose–response relationship between outdoor exposure and reduced axial elongation. Interventions that increased outdoor exposure by 40–80 min per day were associated with significant reductions in axial elongation [3].

Variations in treatment response have been reported among different ethnic groups; however, the protective effect of outdoor exposure on refractive development appears to be broadly consistent across populations. However, the evidence remains moderate due to variability in exposure measurement. Current consensus guidelines recommend at least 2 h of outdoor activity daily as a primary preventive strategy [3].

##### Near Work, Accommodation, and Digital Behavior

Recent longitudinal studies have improved our understanding of the role of near-work activities in myopia development and progression. Although overall screen time is inconsistently associated with myopia, factors such as viewing distance and accommodative stability are more critical. A 2025 population-based study found that accommodative instability and prolonged near-work activity were independently associated with an increased risk of myopia onset [4].

Emerging evidence indicates that increased digital screen time is associated with a greater risk of myopia. The risk appears relatively low at exposure durations below 1 h per day but increases substantially when daily screen use approaches or exceeds 4 h. These findings could guide future research and shape educational and public health policies to address the myopia epidemic [14].

The 20–20–20 rule has gained increasing support as a behavioral strategy for reducing accommodative stress and visual fatigue to reduce accommodative lag and eye fatigue, thereby helping prevent axial elongation [4,15].

Greater degrees of myopia have been reported in individuals presenting with ocular surface symptoms and in individuals with noted eye-rubbing behaviors. Although evidence remains limited, eye-rubbing behavior has been proposed as a potential contributing factor in myopia development, possibly through alterations in ocular biomechanics [16]. Although the association between keratoconus and allergic disease is well established, recent studies have also explored a potential relationship between allergic conditions and myopia development [17].

Ocular surface alterations, including tear film instability and reduced tear secretion, have been associated with structural and biomechanical changes of the cornea, highlighting the complex interaction between ocular surface status and refractive or biometric parameters [18].

##### Indoor Lighting and Spectral Composition (Table 1)

Recent evidence suggests that light exposure characteristics may influence both the development and progression of myopia. Both experimental and epidemiological studies have shown that low-frequency fluorescent flicker may cause myopic changes, whereas exposure to violet light (360–400 nm) may activate retinal dopaminergic pathways that inhibit axial elongation [4,15].

**Table 1:** Evidence-based recommendations for environmental and behavioral strategies for myopia prevention and control.

Recommendation	Evidence Type	Key Findings	Confidence
≥2 h/day outdoor exposure	Systematic review and meta-analysis of RCTs [9]	Reduced incidence of myopia and slower axial elongation dose-response effect observed with 40–80 additional min/day outdoors	Moderate-High
Regular breaks during near work (20–20–20 rule)	Cohort and observational studies [4,15]	Reduced accommodative stress and visual fatigue; limited evidence for direct reduction of axial elongation	Moderate
Limit prolonged screen exposure (>4 h/day)	Dose-response meta-analysis [14]	Increasing screen time associated with progressively greater myopia risk	Moderate
Monitor accommodative instability and excessive near work	Population-based cohort study [4]	Associated with increased risk of myopia onset	Moderate
Improve classroom lighting and daylight exposure	Observational and experimental studies [15]	Lower incidence of myopia reported in naturally illuminated environments	Low-Moderate
Address ocular surface disease and discourage eye rubbing	Observational studies [16,17,18]	Potential reduction of biomechanical and inflammatory contributors to myopia development and progression	Low

Classrooms equipped with lighting systems that more closely replicate natural daylight have been associated with a reduced incidence of myopia in school-aged children [15].

### 3.3. Ocular Biometric and Structural Predictors

#### 3.3.1. Axial Length and AL/CR Ratio

Axial length remains the strongest predictor of both myopia onset and progression. Updated pediatric nomograms incorporating axial length, the AL/CR ratio, and uncorrected visual acuity demonstrate high accuracy in identifying premyopia [19].

Longitudinal population-based studies indicate that shifts in axial length distribution are occurring at increasingly younger ages, even in Western populations, before changes in refractive error occur [8]. Axial elongation in myopic children is more rapid at younger ages and gradually slows over time, apparently independent of age at onset. At age six, axial length percentiles were similar among all racial and ethnic groups. Although females exhibited axial lengths that were approximately 0.4–0.5 mm shorter than those of males, they demonstrated a higher prevalence of myopia across age-specific percentiles [20].

#### 3.3.2. Choroidal Biomarkers and Posterior Pole Remodeling

Choroidal thinning, stromal regression, and reduced vascularity appear to precede and predict axial elongation, according to the most recent studies published in 2024 and 2025, involving high-resolution OCT and OCT angiography. Structural analyses demonstrate stromal regression and reduced choroidal volume correlating with increasingly severe myopia [21].

Eyes with posterior staphyloma exhibit characteristic alterations in choroidal perfusion and posterior pole curvature, supporting a biomechanical deformation model of high myopia progression [22]. Additionally, macular ridges have emerged as a novel biomarker associated with increasing axial length and age [21].

Single-point measurements of choroidal thickness do not fully characterize choroidal structure. To better understand these changes, the choroidal area (CA) should be measured within a 3 mm circle around the fovea, which often shows a significant reduction in pathologic myopia. The choroid’s essential role in retinal health is well-established. Binarization was used to separate blood vessels from stroma in OCT images, allowing measurement of the vascular and matrix areas; both decreased in myopia, while axial length increased. These findings suggest that stromal reduction may exceed vascular loss during disease progression [23,24].

#### 3.3.3. Optic Nerve Head Adaptation

Longitudinal pediatric imaging studies show that Bruch’s membrane opening-minimum rim width (BMO-MRW) increases with axial elongation, indicating adaptive remodeling of the tissues around the optic nerve rather than glaucomatous damage. This parameter may serve as an early structural biomarker of ocular growth patterns [25].

Peripapillary atrophy is frequently observed in myopic eyes and is not specific to myopic optic neuropathy (MON). The International Myopia Institute defines myopia-associated glaucoma (MAG) as loss of the neuroretinal rim and enlargement of the optic cup in highly myopic eyes with a large optic disc or peripapillary delta zone, even if intraocular pressure is normal [26,27]. Myopic optic neuropathy is not limited to glaucoma-like optic nerve injury. In a recent study, Chen et al. described MON as myopic eyes with non-glaucomatous RNFL and visual field loss, marked by superonasal or nasal RNFL thinning and inferotemporal field defects. Other features, such as optic nerve head size and shape, gamma zone, ganglion cell changes, and microvascular alterations, are clinically relevant but are not yet included in the formal definition [26]. Table 2 summarizes the principal ocular biometric and structural predictors of myopia progression.

### 3.4. Molecular and Biological Mechanisms

#### 3.4.1. Growth Factor and Hypoxia Pathways

Recent analyses of aqueous humor samples have demonstrated that HB-EGF and EGF concentrations increase with axial length, whereas VEGF-A levels decrease, suggesting alterations in hypoxia-related signaling pathways and extracellular matrix remodeling in elongated eyes [10,24].

Ultra-wide-field OCTA studies indicate that the posterior staphyloma is accompanied by disturbed perfusion signatures and altered Gaussian curvature of the posterior eye wall. These findings support the hypothesis that choroidal hypoperfusion and scleral hypoxia may function as important triggers of growth-regulating pathways involved in axial elongation [22].

#### 3.4.2. Dopaminergic and Phototransduction Signaling

Dopamine is believed to regulate ocular growth through multiple interconnected mechanisms: it can act directly on dopamine receptors within visual pathways to influence myopia development, or activity within visual pathways may influence dopamine release and signaling, thereby modulating ocular growth and myopia progression [28].

Violet light-induced dopamine release is considered one of the principal mechanisms underlying the protective effect of outdoor exposure against myopia development. Recent experimental studies suggest that dopaminergic signaling may influence scleral fibroblast activity and extracellular matrix remodeling, reducing excessive axial growth [15,29].

#### 3.4.3. Scleral Extracellular Matrix Remodeling

Genetic and transcriptomic studies have demonstrated dysregulation of genes involved in collagen synthesis, extracellular matrix cross-linking enzymes (including members of the LOXL family), and matrix metalloproteinases within the myopic sclera [30]. Abnormal biomechanical forces activate mechanotransduction pathways, initiating a feedback loop in which cyclic stretching increases the expression of miR-21 and miR-34a. These microRNAs suppress antifibrotic regulatory pathways, thereby promoting myofibroblast differentiation, protease secretion, extracellular matrix degradation, and progressive scleral remodeling and protease secretion that promote ECM degradation and ectatic distortion. MicroRNAs (miRNAs) can modulate extracellular matrix gene expression and fibroblast behavior within ocular tissues, suggesting a potential mechanism linking molecular signaling to scleral remodeling [31]. Collectively, these molecular alterations reduce the biomechanical integrity of the sclera, thereby increasing susceptibility to axial elongation. Myopia progression is also characterized by scleral thinning, decreased collagen synthesis, and heightened extracellular matrix degradation [11].

To date, most investigations have focused on whole-sclera analyses to understand how ocular elongation contributes to scleral thinning and biomechanical stretching. However, this approach may have limited ability to identify localized, cell-specific molecular signaling pathways involved in myopia development [32].

Additional studies suggest that scleral hypoxia contributes to myopia development and that therapies targeting hypoxia-related pathways may help slow disease progression. These findings support the hypothesis that reducing scleral hypoxia may represent a promising therapeutic strategy for myopia control [32]. Table 3 summarizes the molecular and biological mechanisms involved.

## 4. Genetic Contributions to Myopia

### 4.1. Contemporary Genetic Architecture

Recent large-scale genetic studies have confirmed that myopia is a highly polygenic disorder, with more than 400 susceptibility loci identified through genome-wide association studies (GWAS), whole-exome sequencing, and family-based investigations. These loci are involved in biological pathways associated with ocular growth regulation, extracellular matrix remodeling, neuronal signaling, and retinal phototransduction [33].

Recent syntheses of global GWAS datasets indicate that the strongest genetic associations are linked to axial length regulation rather than refractive error itself. These findings further support axial elongation as the principal structural mechanism underlying myopia development and progression [34].

Genetic factors play a substantial role in refractive development, with heritability estimates ranging from approximately 50% to more than 90% in twin studies. Twin studies consistently demonstrate significantly higher concordance rates among monozygotic twins than among dizygotic twins, indicating a strong genetic influence on both refractive error and axial length [11].

### 4.2. Polygenic Risk Scores and Risk Stratification

Recent clinical studies suggest that polygenic risk scores (PRSs) may improve the prediction of early-onset and high myopia when combined with biometric parameters and parental history [33].

In both Asian and European pediatric cohorts, incorporation of PRSs significantly improved predictive accuracy for high myopia, with AUC values around 0.75 to 0.80, particularly in children exposed to intensive educational demands. Importantly, these findings support the potential use of PRSs to guide early intervention strategies, emphasizing their value in preventive risk stratification rather than deterministic prediction of disease outcomes [35,36].

### 4.3. Rare Variants and Early-Onset High Myopia

Advances in trio-based exome sequencing have identified de novo and rare pathogenic variants associated with severe early-onset high myopia. These include genes related to scleral collagen cross-linking, retinal development, and ciliary and photoreceptor signaling. These findings may explain a subset of cases characterized by rapid progression despite limited environmental risk exposure, supporting the role of genetic testing and counseling in atypical or syndromic forms of myopia [37]. Flitcroft et al. identified numerous candidate genes involved in refractive development, highlighting pathways such as ECM remodeling, glycosylation, and axon guidance [25].

### 4.4. Gene-Environment Interaction

Studies conducted in both Europe and Asia indicate that educational exposure and prolonged near-work activity may amplify genetic susceptibility to myopia, whereas outdoor exposure appears to attenuate genetic risk. A European meta-analysis reported that myopia-associated single nucleotide polymorphisms (SNPs) exert weaker effects among individuals with lower educational exposure and stronger effects in those with higher education levels. Additionally, increased outdoor exposure may partially mitigate the effects associated with elevated polygenic risk [35,38].

Emmetropization during early childhood may partially compensate for both genetic and environmental risk factors; however, this protective mechanism appears to diminish once axial elongation exceeds age-appropriate developmental thresholds [39].

Taken together, current evidence supports a multifactorial model of myopia development in which genetic predisposition interacts dynamically with environmental exposures to determine individual susceptibility, age of onset, and disease progression [38,39].

## 5. Follow-Up and Monitoring Strategies

### 5.1. Risk-Based Screening

Nomogram-based prediction models incorporating axial length (AL), the axial length-to-corneal radius ratio (AL/CR), uncorrected visual acuity (VA), and non-cycloplegic spherical equivalent refraction (SER) have demonstrated high sensitivity and specificity for identifying children at risk of premyopia [4].

Updated European consensus recommendations advocate targeted screening of children with known risk factors, particularly those with one or both myopic parents (especially if both parents are affected), early axial elongation, limited outdoor activity, or high educational pressure. Population-based datasets, including national conscript registries and school-based screening programs, provide valuable normative data for axial length monitoring and risk stratification [10,40].

The Myopia Progression Risk Assessment Score (MPRAS) has been proposed as a clinical decision-support tool for identifying individuals at increased risk of myopia progression. This model considers factors such as age of onset and presentation, number of myopic parents, spherical equivalent, one-year refractive change, peripheral refraction, near phoria, accommodative error, and time spent near work and outdoor activities. In prospective validation studies, the MPRAS model demonstrated a sensitivity of 94% and a specificity of 60% for predicting myopia progression [35].

### 5.2. Monitoring Intervals

For patients with suspected premyopia, axial length and refractive status should be assessed every six months. A minimum of two hours of outdoor activity per day should be encouraged as part of preventive lifestyle management [5,8,15]. If progression is documented, typically defined as axial elongation exceeding 0.20 mm within six months, follow-up evaluations at intervals of three to four months should be considered [6,10].

Patients receiving active myopia-control interventions should undergo axial length measurements every six months and cycloplegic refraction at least annually. OCT and OCTA may additionally be used to monitor choroidal changes and treatment response [22,41,42].

Adequate cycloplegia is essential for accurate refractive assessment in children because of their strong accommodative capacity. One study showed that, in school-aged kids non-cycloplegic refraction overestimated myopia by an average of 0.65 D compared with cycloplegic measurements [43,44].

### 5.3. Monitoring for Pathologic Myopia

Recent longitudinal studies indicate that excessive axial length, lacquer cracks, and progressive choroidal thinning are associated with an increased risk of myopic macular neovascularization (mMNV) and other degenerative complications [45]. Lim et al. reported that the presence of lacquer cracks significantly increased the risk of second-eye involvement by mMNV over a 10-year follow-up period [3]. Structural biomarkers, including macular ridges and alterations in posterior pole curvature, are increasingly recognized as indicators of progressive biomechanical remodeling in highly myopic eyes [21].

Previous studies and consensus classifications have proposed refined grading systems for myopic maculopathy based on the long-term risk of vision-threatening complications. Lacquer cracks, myopic neovascularization, and Fuchs’ spots are classified as additional lesions associated with an increased risk of central vision loss [46]. Fluorescein angiography remains an important imaging modality for detecting choroidal neovascularization in highly myopic eyes. Early scans can reveal transmission defects or RPE atrophy in the macula and around the optic disc. Spectral-domain optical coherence tomography (SD-OCT) remains the preferred modality for monitoring myopic choroidal neovascularization because it is non-invasive, widely available, and allows quantitative assessment of disease activity, although fluorescein angiography and indocyanine green angiography may offer greater sensitivity for lesion detection in selected cases [47].

## 6. Therapeutic Interventions

### 6.1. Behavioral Interventions

Current evidence suggests that accommodative stability may play a more important role in myopia progression than total screen exposure alone. Although total screen time has not been consistently associated with myopia development, screen use that incorporates regular distance-viewing breaks may reduce accommodative stress and visual fatigue by reducing accommodative effort [14]. Behavioral strategies such as the 20–20–20 rule may reduce accommodative lag and visual fatigue and could indirectly contribute to slowing myopia progression [4].

Recent studies, including the meta-analysis of Martinez-Perez et al., have shown that following approximately two hours of outdoor daylight exposure, transient choroidal thickening has been observed, and the total retinal thickness increased and stayed stable at the follow-up. These findings suggest that light exposure may induce transient structural changes within the retina and choroid that could influence ocular growth regulation [9,36,48].

### 6.2. Optical Interventions

A 2025 network meta-analysis identified several highly effective optical interventions for reducing axial elongation in children with myopia: orthokeratology (OK), highly aspherical lenslets (HAL), defocus-incorporated multiple-segment (DIMS) lenses, and CARE-type annular defocus spectacles. HAL lenses ranked among the top-performing spectacles for axial-length control, and DIMS lenses have also demonstrated clinically meaningful reductions in axial elongation, have a good safety profile, and the evidence from multi-year randomized controlled trials (RCTs) promotes them as an established evidence-based treatment [38,39]. Several studies suggest that myopic defocus may enhance choroidal blood flow and thereby influence retinal and scleral signaling pathways involved in ocular growth regulation, and this mechanism may help inhibit axial elongation through modulation of choroidal perfusion and scleral signaling pathways [49]. HAL lenses generate peripheral defocus patterns that may influence retinal signaling pathways involved in ocular growth regulation. Studies generally show efficacy equal to or greater than DIMS in slowing axial growth and currently represent a clinically applicable therapeutic method [50]. Bao et al. reported a clinically significant hyperopic shift in 20% of participants using HAL lenses compared with 4% of those using slightly aspherical lenses (SAL). Additionally, a decrease in AL was seen in 26% of the HAL group and 5% of the SAL group. Comparable changes were not observed among participants wearing single-vision lenses [51]. Spectacle-based defocus designs are increasingly used because of their favorable safety profile and clinical efficacy [30,52]. CARE and CARE-S spectacle designs have been reported to reduce axial elongation by approximately 40% compared with conventional single-vision spectacles. Earlier research employing various optical myopia-control approaches has consistently indicated that treatment responses depend on age, with more pronounced effects noted in younger patients [39,53].

Network meta-analyses support the efficacy of orthokeratology (OK) in reducing axial elongation, with treatment effects comparable to those reported for low-dose atropine (0.01–0.05%) over a two-year period. In controlled clinical settings, the incidence of serious complications, including infectious keratitis, appears to remain low when appropriate patient selection and monitoring protocols are followed [10,54]. The MESOK study demonstrated that Breath-O-Correct orthokeratology lenses effectively reduced myopia progression in children, reducing AL elongation by 0.17 and 0.22 mm over 12 and 24 months, respectively, compared to SVL. No significant adverse effects on safety parameters were observed, except for a reduction in central corneal thickness, aligning with OK lens effects and previous reports [55,56]. In studies evaluating Asian populations, orthokeratology reduced axial elongation by about 50% over 2 years, with an average change of 0.3 mm compared with 0.6 mm in control groups, equating to roughly 0.5 D of refractive change. OK is known as an established myopia-control treatment, widely used in clinical practice. Also, it is mentioned that rebound axial elongation may occur following treatment discontinuation or modification [57]. Material biocompatibility is essential for maintaining ocular surface health and long-term treatment tolerability during contact lens wear. A notable characteristic of contact lens materials is their ability to maintain physical properties after being subjected to different external conditions. Silicone-hydrogel lens materials demonstrate water-dependent biomechanical properties that may influence flexibility and ocular compatibility [58]. Orthokeratology reduces the risk of amblyopia in children with myopic anisometropia and improves their visual acuity and quality of life [59,60]. The patient’s pupillary diameter is an important factor in choosing the type of contact lenses. For students with pupils measuring 4.0 mm or less, daytime lenses are more effective than OK lenses because the back optical zone diameter of the OK lenses affects the distance vision clarity. The impact of soft contact lenses is more pronounced in small pupils, where the therapeutic area is located within 5.0 mm [61].

According to the World Society of Pediatric Ophthalmology and Strabismus Myopia Consensus Statement 2025, the interventions for which evidence of efficacy remains insufficient include under-correction of myopia, pinhole or blue light blocking glasses, standard and positively aspherized progressive addition lenses, bifocal glasses, soft contact lenses, and rigid gas permeable lenses worn during the day [62].

Soft contact lenses serve as a practical non-pharmacological therapeutic approach for reducing myopia progression in children by inducing peripheral myopic defocus while ensuring clear central vision. Research from a systematic review shows that dual-focus, multifocal, and extended-depth-of-focus soft contact lenses significantly reduce axial elongation and refractive progression compared with single-vision corrections, exhibiting effective results and a satisfactory safety profile when proper follow-up is ensured [54]. Long-term studies on dual-focus soft contact lenses have demonstrated sustained treatment effects and a good safety profile in pediatric populations [54,60]. Also, the beneficial effects of multifocal soft contact lenses have been consistently reported across multiple studies [54,60]. Walline, et al., in the BLINK randomized clinical trial investigated whether soft multifocal contact lenses could reduce the progression of myopia in children between 7 to 11 years of age. Researchers followed 294 children for three years, revealing that those using high add power multifocal lenses experienced the slowest myopia progression and the minimal eye elongation. The authors found that high add power multifocal contact lenses represent a potentially effective approach for myopia management in children, though additional studies are required to determine the long-term clinical benefits and outcomes [63]. Extended-Depth-of-Focus (EDOF) soft contact lenses are considered a promising contemporary intervention in reducing myopia progression and slowing axial elongation, with increasing evidence supporting their use in long-term myopia management programs [54,64].

The 2025 consensus from the World Society of Pediatric Ophthalmology and Strabismus acknowledges soft multifocal contact lenses as a validated evidence-based intervention that can effectively reduce myopia progression and should be considered in children at risk of developing high myopia [62].

Table 4 provides a concise overview of selected studies that address optical interventions.

### 6.3. Pharmacological Interventions

#### 6.3.1. Atropine

Substantial evidence supporting the use of atropine for myopia control has accumulated over the past two decades, when the ATOM1 study found that nightly administration of 1% atropine reduced myopia progression by approximately 77% over two years, with minimal axial elongation compared with the control group. Further, the ATOM2 trial showed that 0.01% Atropine remained effective while being associated with relatively few adverse effects, such as a slight reduction in accommodation [64,65]. The results from the MOSAIC trial by Pärssinen et al. show that 0.01% Atropine stabilizes choroidal thinning compared with placebo [15]. Evidence suggests that low-dose atropine (0.01–0.05%) remains an important therapeutic option for myopia control. Recent studies in Europe and Asia show that effectiveness depends on the dose, with low concentrations providing a good safety profile, and suggest that choroidal stabilization may serve as a potential biomarker of therapeutic response. Atropine 0.01% is clinically used, but is no longer considered the optimal evidence-based concentration [66]. While higher concentrations, such as 0.05–0.1%, may be more effective, higher concentrations may compromise treatment adherence because of dose-dependent adverse effects like photophobia and near-vision blur [30,45,67]. The long-term findings from the LAMP study revealed that, over 3 years, participants in the taper group, who had gradually reduced Atropine concentrations, experienced less myopia progression compared to the stop group, especially in younger children with higher myopia [67].

Several studies suggest that combination treatment strategies may provide greater efficacy. Guemes-Villahoz et al. in the ASPECT (Atropine and Spectacle Lens Combination Treatment) randomized trial, 0.025% atropine combined with DIMS spectacle lenses significantly reduced axial elongation over 12 months (0.07 ± 0.16 mm) compared with 0.025 atropine plus single-vision spectacles (0.18 ± 0.16 mm) [68]. A retrospective study showed that adding 0.01% or 0.125% atropine to DIMS lenses enhanced the reduction of refractive progression and axial elongation compared to DIMS lenses alone, with no extra advantage from the higher concentration of atropine. These results indicate a combined effect of using pharmacological and optical treatments for managing pediatric myopia [69]. Combination therapy is applicable in selected patients, clinically used in fast progressors, though protocols continue to evolve [59].

Exosomes, a subtype of extracellular vesicles ranging from 30 to 150 nm in diameter, have garnered attention as potential drug carriers because of their biocompatibility, stability, low toxicity, and reduced immunogenicity. Their innate capacity to carry proteins, lipids, and nucleic acids allows for effective delivery of therapeutic substances to the eye tissues. Recent reviews have evaluated advances in exosome-based ocular drug delivery systems and current clinical trials, emphasizing their ability to enhance the treatment of eye conditions and support clinical application [70].

#### 6.3.2. Low-Level Red-Light Therapy

Low-level red-light (LLRL) therapy is an emerging non-invasive intervention currently under investigation for myopia control [71]. LLRL procedure uses low-intensity (650 nm) red light, applied for 3 min twice daily, 5 days a week [72]. Improvements in choroidal thickness, vascularity, and luminal area have been reported following LLRL therapy and appear to correlate with axial length and spherical equivalent progression. Preliminary findings suggest that LLRL therapy may improve choroidal perfusion and structural parameters and decrease myopia-related degeneration [73]. Recent randomized controlled trials have examined the use of repeated low-level red-light therapy to slow myopia progression in children. Several randomized controlled trials have reported significant reductions in myopia progression, although longer-term safety data remain limited [74,75]. Although the precise mechanisms remain incompletely understood, red-light exposure may enhance choroidal perfusion and modulate pathways involved in ocular growth regulation and slow myopia progression. The choroid supplies approximately 85% of the blood flow to the outer retina. Repeated Low-Level Red-Light Therapy (RLRL) is emerging and many studies, primarily from China, report substantial reductions in myopia progression and axial elongation [75,76].

Current literature suggests that LLRL therapy may reduce axial elongation; however, long-term retinal safety data are still limited, so cautious implementation is advised [30]. Schmidt et al. found that LLRL therapy, through a photo biomodulation mechanism, may effectively slow axial length progression, but ongoing retinal monitoring remains advisable. Most published studies have relatively short follow-up periods, typically between 6 and 24 months. Therefore, the long-term efficacy, durability of treatment effects, and safety profile of (LLRL) remain poorly established. Emerging evidence suggests the possibility of rebound progression following discontinuation of LLRL therapy, raising concerns about the sustainability of treatment benefits and the optimal duration of therapy. In addition, concerns about rebound progression after treatment discontinuation warrant further investigation in large-scale multicenter studies with extended follow-up [9,10].

## 7. Discussion

Contemporary myopia research is increasingly shifting toward prevention, precision medicine, and personalized treatment strategies, with particular emphasis on axial length monitoring and individualized risk assessment. Recent reviews have highlighted the need for standardized approaches to measuring axial elongation and for expanding research beyond predominantly Asian pediatric populations [10]. In a cohort study, Kang et al. demonstrated that deep-learning models incorporating limited baseline clinical data could accurately predict the risk of both myopia and high myopia. These findings suggest that artificial intelligence–based predictive models may facilitate large-scale screening programs and support earlier intervention, particularly in resource-limited settings [77].

Innovative optical interventions, including advanced spectacle lens designs, continue to demonstrate promising efficacy and are increasingly being evaluated across diverse clinical settings and study designs []. Population-level prevention strategies increasingly focus on optimizing outdoor exposure and light-related interventions through evidence-based dose–response recommendations, with growing interest in understanding how the intensity, duration, timing, and adherence to these interventions influence axial elongation []. Advanced imaging technologies and emerging biomarkers may improve our understanding of treatment response and facilitate the identification of individuals most likely to benefit from specific interventions. Choroidal parameters derived from OCT and OCT angiography (OCTA) are increasingly being investigated as monitoring tools and potential surrogate biomarkers of treatment response [22,78]. Molecular investigations of aqueous humor biomarkers may identify growth factor profiles associated with axial elongation and could facilitate the development of novel pharmacological targets beyond atropine therapy [29].

## 8. Future Directions

Artificial Intelligence and Myopia Management. Artificial intelligence (AI) has emerged as a promising tool in ophthalmology, with potential applications in predictive modeling, clinical decision support, disease screening, and therapeutic decision-making. Numerous studies have demonstrated the utility of AI-based classification algorithms in the detection of glaucoma, diabetic retinopathy, and other retinal diseases. In myopia research, AI models can integrate structured clinical, biometric, imaging, genetic, and environmental data, including axial length, corneal curvature, retinal imaging parameters, genetic susceptibility markers, screen exposure, and outdoor activity patterns [79,80]. These multidimensional datasets may be incorporated into predictive models for individualized risk assessment, disease monitoring, and treatment planning. Machine-learning frameworks may facilitate early detection of myopia, support personalized intervention strategies, and enhance clinical decision-making through data-driven risk prediction models. The study by Lin, Long, and Ding demonstrated the feasibility of using machine-learning algorithms to predict myopia onset and progression from electronic medical record (EMR) data, highlighting the potential of AI-driven risk stratification [81]. AI-based models incorporating axial elongation, refractive status, demographic variables, and lifestyle parameters have demonstrated promising predictive performance compared with conventional risk-factor-based approaches in predicting myopia onset and progression [81]. The study provides empirical support for extending optimized machine learning models (such as SVM combined with DE) to improve risk prediction and support personalized myopia management. As AI technologies continue to evolve, their role is expected to expand beyond screening toward clinical decision support systems capable of optimizing efficacy, safety, and cost-effectiveness within individualized myopia management strategies [82].

Recent genetic analyses have identified numerous loci associated with myopia susceptibility and ocular growth regulation. Gene-expression analyses have reported reduced CPNE1 expression in myopic scleral tissue. These findings suggest that CPNE1 may contribute to pathways involved in scleral remodeling and myopia pathogenesis via regulation associated with the cell cycle and improvement of scleral fibrotic remodeling, potentially representing a future therapeutic target [83].

Experimental strategies targeting scleral cross-linking, extracellular matrix remodeling, hypoxia-related pathways, and dopaminergic signaling represent promising areas of investigation; however, robust efficacy and safety data are required before clinical implementation [82].

## 9. Conclusions

The rapidly increasing global prevalence of myopia represents one of the most significant public health challenges in contemporary ophthalmology. Current epidemiological evidence indicates that the rising prevalence of myopia cannot be explained solely by genetic predisposition and is strongly influenced by environmental, behavioral, and lifestyle-related factors. Evidence accumulated over recent decades consistently demonstrates strong associations between intensive educational exposure, prolonged near-work activities, limited outdoor exposure, and the onset of myopia, with consistent epidemiological trends across different populations and time periods.

Advances in myopia control over the past decade have shifted clinical management from simple refractive correction toward active prevention of excessive axial elongation and long-term ocular complications.

Effective myopia management begins with a comprehensive clinical assessment that includes age at onset, family history, outdoor exposure, near-work behavior, refractive status, and biometric measurements. This assessment should be complemented by cycloplegic refraction, axial length measurement using optical biometry, fundus examination, and binocular vision evaluation.

Future clinical strategies are likely to incorporate genetic risk profiling, advanced imaging biomarkers, artificial intelligence-assisted prediction models, and individualized treatment algorithms, thereby facilitating precision-medicine approaches to myopia management. Ultimately, early identification of at-risk individuals and timely implementation of evidence-based interventions remain the most effective strategies for reducing the lifelong burden of myopia and preventing vision-threatening complications.

## Figures and Tables

**Table 2:** The synthesis of ocular biometric and structural predictors and their clinical significance.

Subcategory	Factor/Biomarker	Key Point	Clinical Significance
Axial Length & AL/CR	Axial length	Strongest prediction of myopia onset and progression [19,20]	Early risk identification [19]
	AL/CR ratio	Included in modern pediatric nomograms [19]	Improves detection of pre-myopia [19]
	Early axial growth	Occurs at younger ages, before refractive changes [25]	Supports early intervention [25]
	Sex differences	Females have shorter AL but higher myopia prevalence [13,20]	Suggests additional contributing factors [20]
	Growth rate	Faster in early childhood then slows [20]	Important for monitoring progression [20]
Choroid & Posterior Pole	Choroidal thinning	Precedes axial elongation [21]	Early biomarker of progression [21]
	Stromal & vascular reduction	Associated with severe myopia [23,24]	Indicates structural degeneration [23,24]
	Posterior staphyloma	Alters perfusion and curvature [22]	Biomechanical deformation model [22]
	Macular ridges	Correlates with age and axial length [21]	Emerging biomarker [21]
	Choroidal area (CA)	Reduced in pathologic myopia [23,24]	More comprehensive than single-point thickness measurements [23,24]
	OCT binarization	Separates vascular and stromal components [23,24]	Shows greater stromal loss [23,24]
Optic Nerve Head	BMO-MRW	Increases with axial elongation [25]	Reflects adaptive remodeling, not glaucoma [25]
	Peripapillary atrophy	Common in myopic eyes [26,27]	Not specific for neuropathy [26]
	MAG (myopia-associated glaucoma)	Rim loss and optic cup enlargement [26,27]	Can occur with normal IOP [26,27]
	Myopic optic neuropathy (MON)	RNFL thinning and visual field defects [26,27]	Extends beyond glaucoma-like damage [26,27]
	Additional features	Gamma zone, GCC, microvascular changes [26]	Clinically relevant but not formally defined [26]

**Table 3:** Molecular and biological mechanisms implicated in myopia development and progression and their principal findings.

Section	Key Mechanisms	Main Findings
Aqueous Humor Changes and Ocular Perfusion	Altered growth factors (HB-EGF, EGF ↑; VEGF-A ↓); impaired choroidal perfusion; scleral hypoxia [10,24]	Increased axial length correlates with higher HB-EGF and EGF and lower VEGF-A; OCT-A shows disturbed perfusion and structural deformation (posterior staphyloma, altered curvature) [22,24]
Dopaminergic and Phototransduction Signaling	Dopamine acts on retinal receptors; violet light stimulates dopamine production [28]	Dopaminergic signaling reduces excessive axial growth by influencing scleral fibroblasts; outdoor light exposure increases dopamine [28]
Scleral Extracellular Matrix Remodeling	Dysregulation of collagen synthesis, LOXL cross-linking enzymes, and metalloproteinases [30]	Leads to weakened scleral structure, thinning, reduced collagen, and increased ECM degradation [30]
Scleral Hypoxia and Molecular Signaling	Reduced oxygen supply alters scleral metabolism and signaling pathways [32]	Hypoxia contributes to myopia development; anti-hypoxia drugs can slow progression [32]

**Table 4:** Representative studies evaluating the efficacy of optical interventions for myopia control.

Category	Intervention	Key Findings	Clinical Relevance
Spectacle-based lenses	HAL	Among most, significant AL reduction [39,40]	Effective noninvasive treatment option [39,40]
	DIMS	Proven reduction in axial elongation [39,40]	Widely adopted in clinical practice [38,39]
	CARE/CARE-S	~40% less AL elongation vs. SVL [39]	High efficacy [39,53]
	SAL	Modest effect [51]	Less effective alternative [51]
	SVL	No impact on AL [51]	Control standard [51]
Rigid contact lenses	Orthokeratology (OK)	~50% reduction in AL over 2 years [57]	Comparable to low-dose atropine [10,54]
	Breath-O-Correct OK	Reduced AL by ~0.17–0.22 mm [55,56]	Safe in children [55,56]
Soft contact lenses	Dual-focus	Slow myopia progression and axial elongation compared with single-vision correction [54,60]	Strong level of evidence. Suitable as a first-line option in children based on their efficacy, safety, and ease of use [61]
	Multifocal center-distance	Reduce refractive progression and ocular axial growth [54,60,63]	Effective therapeutic option. Requires regular monitoring and individualized treatment planning [59,61,63]
	Extended-depth-of-focus (EDOF)	Associated with slower myopia progression and reduced axial elongation while maintaining good visual performance and tolerability [54,64]	A promising contemporary intervention with increasing evidence supporting its use in long-term myopia management programs [64]

## Data Availability

Data sharing is not applicable to this structured narrative review, as no new datasets were generated or analyzed.
